# Application and evaluation of knowledge graph embeddings in biomedical data

**DOI:** 10.7717/peerj-cs.341

**Published:** 2021-02-18

**Authors:** Mona Alshahrani, Maha A. Thafar, Magbubah Essack

**Affiliations:** 1 Department of Computer Science and Engineering, Jubail University College, Jubail, Saudi Arabia; 2 Computer, Electrical and Mathematical Sciences and Engineering Division (CEMSE), Computational Bioscience Research Center (CBRC), King Abdullah University of Science and Technology (KAUST), Thuwal, Saudi Arabia; 3 College of Computing and Information Technology, Taif University, Taif, Saudi Arabia

**Keywords:** Knowledge graphs, Embeddings methods, Biomedicine, Comparative evaluation, Performance studies, Linked data, Bio-ontologies

## Abstract

Linked data and bio-ontologies enabling knowledge representation, standardization, and dissemination are an integral part of developing biological and biomedical databases. That is, linked data and bio-ontologies are employed in databases to maintain data integrity, data organization, and to empower search capabilities. However, linked data and bio-ontologies are more recently being used to represent information as multi-relational heterogeneous graphs, “knowledge graphs”. The reason being, entities and relations in the knowledge graph can be represented as embedding vectors in semantic space, and these embedding vectors have been used to predict relationships between entities. Such knowledge graph embedding methods provide a practical approach to data analytics and increase chances of building machine learning models with high prediction accuracy that can enhance decision support systems. Here, we present a comparative assessment and a standard benchmark for knowledge graph-based representation learning methods focused on the link prediction task for biological relations. We systematically investigated and compared state-of-the-art embedding methods based on the design settings used for training and evaluation. We further tested various strategies aimed at controlling the amount of information related to each relation in the knowledge graph and its effects on the final performance. We also assessed the quality of the knowledge graph features through clustering and visualization and employed several evaluation metrics to examine their uses and differences. Based on this systematic comparison and assessments, we identify and discuss the limitations of knowledge graph-based representation learning methods and suggest some guidelines for the development of more improved methods.

## Introduction

Knowledge graphs generally refer to a form of knowledge representation that consists of entities and their relation to each other, where heterogeneous knowledge nodes represent entities and labeled edges represent their relation to each other (Färber et al., 2016). Many definitions of knowledge graphs exist in the biomedical field (Ehrlinger & Wöß, 2016). Nonetheless, there are general recommendations and prerequisites of what constitutes a knowledge graph, which mainly emphasizes representing knowledge in a graph structure with labeled edges adhering to explicit, and unambiguous semantics (Ehrlinger & Wöß, 2016; Zhang, 2002).

The relational and semantic representation provided by knowledge graphs is fundamental to logic and inference and has contributed to enhancing the intelligence of Web searches (Raedt et al., 2016; Davis, Shrobe & Szolovits, 1993). A few examples of such publicly available knowledge graphs include FreeBase (Bollacker et al., 2008), and DBpedia (Auer et al., 2007; Lehmann et al., 2015), as well as proprietary knowledge graphs such as the Google knowledge graph (Dong et al., 2014) that have been successfully applied to many real-world applications. To that end, recently, knowledge graphs embedding methods have emerged as an effective and promising paradigm for analyzing and learning from knowledge graphs within and across subject domains. The key idea is to map knowledge graph entities and relations into a low-dimensional vector representation (i.e., an embedding), which preserves its local and global structure and simplify its use in prediction tasks such as link prediction (i.e., knowledge graphs completion), entity classification, and entity resolution (Nickel et al., 2016; Wang et al., 2017).

Utilizing knowledge graph embedding methods to learn, analyze, and visualize biological data is not new. For example, Alshahrani et al. (2017a) demonstrated the integration of biomedical ontologies and linked data in the form of knowledge graphs that were used to predict biological relations. That work has collectively presented several biomedical problems as relations/links prediction. This holistic approach allows the exploitation of existing knowledge about several types of entities and relations to predict the missing ones. In other related work, a specific problem (i.e., relation) was studied in more detail. For example, Alshahrani & Hoehndorf (2018a), Mohamed, Nováček & Nounu (2020) used biomedical knowledge bases to build a knowledge graph consisting of the drugs and their target and used it to infer candidate drugs. Another example is utilizing Knowledge graphs for gene-disease prioritization (Alshahrani & Hoehndorf, 2018b) or predicting disease co-morbidity (Biswas, Mitra & Rao, 2019). For ontologies-based embedding learning, Kulmanov et al. (2019) utilized such embedding techniques to learn from background knowledge accessed through bio-ontologies and showed its successful application through relation predictions that involve entities annotated with ontology classes (Kulmanov et al., 2019; Holter et al., 2019).

Utilizing knowledge graphs in biomedical domains serves many purposes: (1) Knowledge graphs can represent different types of knowledge due to their versatile nature of modeling biological systems through complex interactions between different types of entities. It differs from traditional graph mining techniques due to their limited expressiveness are not able to preserve semantic relations between entities of the graph and can not distinguish between different interactions. For example, in protein-protein interactions networks, traditional graph or network methods can not differentiate between inhibition, activation, or phosphorylation. (2) Knowledge graphs can encompass formal knowledge such as the ones found in bio-ontologies, which makes a distinction between classes and data instances and allows formal inferences to be performed (Alshahrani et al., 2017a; Holter et al., 2019). (3) Knowledge graphs adapt the linked data standards when referring to entities and relations and use (Uniform Resource Identifiers) to facilitate linking and sharing. This property allows multimodal integration of knowledge between different types of knowledge bases and data sources such as all types of networks, biomedical literature, and images.

There are several such methods, and here we establish a systematic study of these methods. To accomplish this task, we surveyed knowledge graph embedding methods, then grouped them into seven categories according to the techniques they employ, the information they contain and the features they encode. We then selected the most representative and successful method of each group for our analyses. To conduct a set of experiments that comprehensively compares the performance of the six state-of-the-art knowledge graph embedding methods in different tasks such as relation/link prediction, clustering, and visualization, we employed different evaluation metrics. We discussed their usage and potential biases in specific tasks. We also provide concluding remarks highlighting the limitations of this work and provide a directive for future works. We make all of our data and evaluation scripts available at https://github.com/monaalsh/kg-embeddings-in-biomedicine.

## Methods for Knowledge Graph Embedding

### Notations

We have used the Semantic Web technology “Resource Description Framework”, more commonly known as RDF, as our graph data model that formally describes the semantics or meaning of information (?). To standardize terminologies, we fixed the RDF terminologies to represent the entities and the relations, given that the aim here is to reuse the RDF graphs and bio-ontologies as our framework that fits a wide range of knowledge graphs embedding methods. In RDF terms, a triple (*s*,*p*,*o*) consists of the subject *s*, the predicate/relation *p*, and the object *o*. The equivalent terminology in knowledge graph embedding methods consider a fact (*h*,*r*,*t*) with the *h* as its head, *t* as its tail entities, and *r* as its relation. Boldface lower-case letters denote the embedding (i.e., low-dimensional vector representations) of subject ${\bf s}$, object entities ${\bf o}$, or relations ${\bf p}$. Upper-case boldface letters denote matrices such as **A**.

### Random walk-based embedding methods

Random walk statistics is a popular proximity measure in graphs. A random walk is a stochastic process of traversing a graph to describe a path consisting of a succession of steps selected uniformly. Implementing random walks in knowledge graphs started with the learning of entities features using edge-weighted paths (Lao & Cohen, 2010; Gardner & Mitchell, 2015; Wang et al., 2016). Lao & Cohen (2010) were the first to implement and evaluate edge-weighted paths in several recommendations tasks. They developed and applied a similar random procedure (Lao, Mitchell & Cohen, 2011) to approximately 500,000 beliefs extracted imperfectly by never-ending language learner (NELL) (Carlson et al., 2010). This method developed in Lao, Mitchell & Cohen (2011) outperformed the Horn-clause learning and inference method used by NELL to determine if text extracted from the web was a fact. That is, Lao, Mitchell & Cohen (2011) formulated a link prediction task that demonstrated excellent results in terms of scalability, and predictive performance achieved was double the precision at rank 100 compared to NELL.

This graph-based representation learning led to the development of several graph embedding methods that utilized random walks statistics to improve feature learning for the node similarity, which showed superior performance in link prediction and node clustering. Of those methods, it is worth singling out Deepwalk (Perozzi, Al-Rfou & Skiena, 2014), the first approach to sample short random walk sequences in a network. DeepWalk generalizes recent advancements in language modeling and unsupervised feature learning from sequences of words to networks. DeepWalk uses local information obtained from a stream of random walks to learn the latent representations of the nodes as well as learn the probability distribution of node co-occurrence by treating the walks as equivalent sentences. These latent representations (i.e., node embeddings) capture the nodes’ neighborhood similarity and community membership. The approach taken to develop Deepwalk that bridges the gap between graph embedding and word embedding was inspired by the well-known neural language model SkipGram (Mikolov et al., 2013). SkipGram is a language model that maximizes the co-occurrence probability among the words that appear within a window, w, in a sentence. It predicts the context words given the target word *w*_t_ by maximizing the average log probability as follows:

(1) $$J = 1/N\sum\limits_{t = 1}^N \sum\limits_{ - c \le j \le c,j \ne } logp({w_{t + j}}|{w_t})$$

where *c* is size of the context window.

Node2vec (Grover & Leskovec, 2016) is a generic version of DeepWalk, but it biases the random walk by different sampling strategies using different parameters. Node2vec aims to learn vector representations that obey two characteristics of the graph neighborhood: homophily and structural equivalence. The first one is related to discovering graph communities (i.e., highly connected nodes are closer to each other), achieved through the Depth-first search. The latter is related to finding nodes that share similar roles in different communities, achieved through Breadth-first search. Both DeepWalk and Node2vec embeddings are being used in the biomedical domain, such as for drug-target interaction prediction, and they were effective in producing the desired results (Zong et al., 2017; Thafar et al., 2020a, 2020b).

An additional random walk-based method, struc2vec (Ribeiro, Saverese & Figueiredo, 2017), is a flexible framework for learning embeddings of node’s structural identity that captures the similarity between nodes in a network which perform similar functions. Structural identity is a symmetry concept used to identify network nodes based on the network structure and their relationship to other nodes. As a first step, struct2vec constructs a multilayer weighted network that encodes the structural similarity between nodes. Each layer k is defined using the nodes’ k-hop neighborhoods. Then, the multilayer graph is used to generate node sequence by a weighted random walk. After that, skip-gram or a similar technique is used to learn the embedding from the context given by the sequence for each node. Nodes with high structural similarity are close to each other in the embedding space. An extension to random walk-based graph embedding is Hierarchical random wALK (HALK) (Schlötterer et al., 2019), which removes a percentage of less frequent nodes from the walks while learning its feature representations to captures the global neighborhood, not just the local neighborhood.

The previous graph embeddings techniques are generally used for unlabeled graphs but can be extended to account for edge information and, therefore, can be applied to RDF graphs, as demonstrated by (Ristoski & Paulheim, 2016). The extension to edge-labeled graphs (i.e., RDF graphs) requires converting the graphs into sequences of entities and relations using graph walks (Perozzi, Al-Rfou & Skiena, 2014) or graph kernels (Yanardag & Vishwanathan, 2015), and then applying a neural language model (Mikolov et al., 2013) to learn low-dimensional vector representation.

Alshahrani et al. (2017a) was the first to successfully extend this procedure to biological knowledge graphs and account for implicit knowledge contained within bio-ontologies through automated reasoning. They then applied these processes collectively to demonstrate the prediction of biological relations (i.e., edges) with prediction accuracy that outperformed state-of-the-art methods.

Despite its success in several prediction tasks, random walks approaches are typically concerned with finding a way to learn structural features that could encode features related to the local neighborhood or global positions of nodes in the graphs with limited incorporation or relation-specific features that identify knowledge graphs. In the following sections, we discuss several methods that formally incorporate semantic information through various distance methods, relation-specific (i.e., different types of relations, taxonomies or hierarchies), and precise information of the knowledge graph.

### Distance-based methods

Distance-based knowledge graph methods represent another class of relational learning, based on the idea that entities are similar if their latent feature vectors are close after applying a relational translation using some distance measures. Such methods infer relations between entities by applying vector operations (i.e., translation) such as subtraction of two embedding vectors in the embedding space. Several methods fall into this category, including TransE (Bordes et al., 2011, 2013), TransH (Wang et al., 2014), TransR (Lin et al., 2015b), and PTransE (Lin et al., 2015a), RotatE (Sun et al., 2018), TorusE, and KGLG (Ebisu & Ichise, 2017, 2019). **TransE** is one of the most representative and widely used as a benchmark. TransE model is described as follows:

Given a triple *(subject, predicate, object)* or simply *(s,p,o)*, it aims to make the sum of the subject and predicate vectors as close as possible to the object vector (i.e., ${\bf s} + {\bf p} \approx {\bf o}$) when *(s,p,o)* holds, and the sum is far away otherwise. This is done based on some distance measure $d({\bf s} + {\bf p},{\bf o})$, which is chosen to be *L*_1_ or *L*_2_ norms. The loss function is the max-margin with negative sampling, and it is defined as minimizing the pairwise ranking loss as follows:

(2) $$\mathcal{L} = \sum\limits_{(s,p,o) \in \mathcal{L}} \sum\limits_{({s}^{\prime},p,{o}^{\prime}) \in {\rm \mathcal{L}^{\prime}}} [{\rm{\gamma}} + d({\bf s} + {\bf p},{\bf o}) - d({\bf {s}^{\prime}} + {\bf p},{\bf {o}^{\prime}})]$$

This loss aims to encourage discrimination between positive triples $\mathcal{L}$ and negative triples $\mathcal{L}^{\prime}$, with *γ* as the margin separating them. Although translational models achieved highly successful predictive and efficient performance in knowledge graphs benchmarks datasets (Bollacker et al., 2008; Miller, 1995), they suffered from several limitations. For example, the TransE models one-to-one relations successfully, but it fails to account for other relational patterns and mapping properties such as one-to-many, many-to-one, and many-to-many. TransH (Wang et al., 2014) addressed this limitation by interpreting the relation as a translating operation on a hyperplane with the normal vector ${{\bf w}_{\bf p}}$, in which both *subject* and *object* entities are projected. Therefore, given the triple (*s*,*p*,*o*), the *s* and *o* entity representations after the projection will be:

(3) $${{\bf s}_ \bot } = {\bf s} - {{\bf w}_{\bf p}}^{\rm T}{\bf s}{{\bf w}_{\bf p}},{{\bf o}_ \bot } = {\bf o} - {{\bf w}_{\bf p}}^{\rm T}{\bf o}{{\bf w}_{\bf p}}$$

The loss is defined with the projected *subject* and *object* representations as follows:

(4) $$\mathcal{L} = \sum\limits_{(s,p,o) \in \mathcal{L}} \sum\limits_{({s}^{\prime},p,{o}^{\prime}) \in {\mathcal{L}^{\prime}}} [{\rm{\alpha}} + d({{\bf s}_ \bot } + {\bf p},{{\bf o}_ \bot }) - d({{\bf {s}^{\prime}}_ \bot } + {\bf p},{{\bf {o}^{\prime}}_ \bot })]$$

TransH also proposed simple techniques for sampling the negatives. Even though TransH solved the relations mapping issues by using relations hyperplanes, it still suffers from other issues. For TransE as well as for the TransH model, the entities and the relations are in the same semantic space *R*, so the similar entity appears in the same entity space. However, each entity can have many aspects, and different relations pay attention to the different aspects of the entity. Thus, TransR (Lin et al., 2015b) is proposed to address this issue by modeling entities and relations in two different semantic spaces (i.e., entity space $ {\mathbb{R}}^d$ and the multiple relations space ${\mathbb {R}}^k$) and performs the translation in the corresponding relation space. The *subject* and *object* entities are first projected into the space specific to the relation using the projection matrix ${{\bf M}_{\bf p}} \in {\mathbb {R}}^{k \times d}$, in which case the resulting vector representations are:

(5) $${{\bf s}_ \bot } = {{\bf M}_{\bf p}}{\bf s},{{\bf o}_ \bot } = {{\bf M}_{\bf p}}{\bf o}$$

Similarity, given the projected entities, the loss is defined as:

(6) $$\mathcal{L} = \sum\limits_{(s,p,o) \in {\rm {\cal S}}} \sum\limits_{({s}^{\prime},p,{o}^{\prime}) \in {\rm {{\cal S}}^{\prime}}} [{\rm{\alpha}} + d({{\bf s}_ \bot } + {\bf p},{{\bf o}_ \bot }) - d({{\bf {s}^{\prime}}_ \bot } + {\bf p},{{\bf {o}^{\prime}}_ \bot })]$$

Although TransR is more expressive and can model different aspects of the entities, it suffers from high modeling complexity introduced by the projection matrix. Furthermore, it is insufficient to build just one single relation vector to perform all translations from subject to object. Thus, CTransR (i.e., cluster TransR) is a variant of TransR which clusters different entity pairs into groups and learns distinct relation vectors for each group. CTransR constraints pairs of entities participating in the same relations to exhibit similar features (Lin et al., 2015b). Additionally, PTransE (Path-based extension of TransE) (Lin et al., 2015a) studies the effect of exploring multi-step relation paths using composition operations such as addition and multiplications between entities instead of only direct relations, and this may encode intricate and high-order inference patterns (Wang et al., 2017).

The general intuition of all the above-mentioned methods is to infer the KG’s connectivity patterns based on the observed facts and relations. These patterns are symmetry/antisymmetry, inversion, and composition, and it is important to model these patterns for link prediction tasks. However, none of these methods can model all these patterns. For example, the TransE model (Bordes et al., 2011), which represents relations as translations, aims to model the inversion and composition patterns but not the other. A recent method, RotatE (Sun et al., 2018), is capable of modeling and inferring all of these relation patterns (i.e., symmetry/antisymmetry, inversion, and composition). The RotatE model maps the entities and relations to the complex vector space and defines each relation as a rotation from the source entity to the target entity. Therefore, given the triple (*s*,*p*,*o*), the *object* vector representation is to be equal to ${\bf o} = {\bf s}{\rm \odot }{\bf p}$, where ${\bf s},{\bf p},{\bf o} \in {\mathbb {C}}$ and $\odot$ denotes the element-wise (Hadamard) product. Additionally, the scoring function for the triple is defined as:

(7) $$score({\bf s},{\bf o}) = ||{\bf s}{\rm \odot }{\bf p} - {\bf o}{||^2}$$

### Rule-based embedding methods

In addition to the structural features discussed in the previous sections, knowledge graphs specifically ontologies have model-theoretic semantics. The ontology TBox contains axioms about classes, such as taxonomic relations, equivalence, or disjointness axioms, which includes the knowledge graph learning objective. For representing hierarchical types, Xie et al. (2016a) proposed Type-embodied Knowledge Representation Learning (TKRL). TKRL follows similar approach to TransE, which additionally considers the hierarchical types as projection matrices for entities, with two type encoders designed to model hierarchical structures. Specifically, TKRL projects the *subject* and *object* into their corresponding hierarchical type-specific spaces using the type-specific projection matrices **M**_**ps**_ and **M**_**po**_ as follows:

(8) $${{\bf s}_ \bot } = {{\bf M}_{{\bf ps}}}{\bf s},{{\bf o}_ \bot } = {{\bf M}_{{\bf p}{{\bf o}_{}}}}{\bf o}$$

**M**_**ps**_ and **M**_**po**_ are designed to handle multiple types, and are defined as the weighted sum of all possible type-specific relations that a *subject* or an *object* can belong to as the following:

(9) $${{\bf M}_{{{\bf ps}}}} = \displaystyle{{\sum\nolimits_{i = 1}^{{n_s}} {{\rm{\alpha}} _i}{{\bf M}_{{{\bf t}_{\bf i}}}}} \over {\sum\nolimits_{i = 1}^{{n_s}} {{\rm{\alpha}} _i}}}$$

where *n*_*s*_ is the number of types, *t*_*i*_ is the type the *subject* can belong to, and α_*i*_ is the corresponding weight. Note that the type can be an ontology class, and in this case, the method can learn type-specific representations of the biological entities associated with a particular class in ontology. Moreover, another recent method, Poincaré Embeddings (Nickel & Kiela, 2017), takes a fundamentally different approach in learning hierarchical representations by embedding them into a hyperbolic space—or more precisely into an n-dimensional instead of Euclidean space. The underlying hyperbolic geometry allows learning parsimonious representations of symbolic data by simultaneously capturing hierarchy and similarity. Poincaré is an efficient algorithm to learn the embeddings based on Riemannian optimization and show experimentally that Poincaré embeddings can outperform Euclidean embeddings significantly on data with latent hierarchies, both in terms of representation capacity and in terms of generalization ability. Knowledge graphs can also include other edge semantics (i.e., relations properties) such as transitivity, asymmetry, and reflexivity. Wang, Wang & Guo (2015) integrate rules into embedding models for KB completion. It introduced an integer linear programing (ILP) approach with the objective function generated from embedding models and the constraints translated from the rules. It first learns the embedding through three KG embedding models namely RESCAL (Nickel, Tresp & Kriegel, 2011) (explained later in tensor-based method section), TRESCAL (Chang et al., 2014) an extension of RESCAL, and TransE (Bordes et al., 2013). Then, it solves the ILP problem by optimizing the normalized scores from the three models under the rules. The incorporation of rules reduces the solution space significantly and enhances the inference of KG completion accuracy. Later, Guo et al. (2016) proposed KALE as a joint model that embeds the KG facts and the logical rules in a unified framework, by reusing the transnational assumption to model the facts and t-norm fuzzy logic to model the logical rules.

Another rule-based KG embedding method, Hierarchical Relation Structure (HRS) (Zhang et al., 2018), which extends the existing KG embedding models TransE, TransH, and DistMult, to learn embedding by leveraging the rich information. According to HRS, the knowledge graph’s relations conform to three layers: relation clusters, relations, and sub-relations, which can fit in the top, the middle, and the bottom layer of three-layer HRS, respectively.

While translational embedding techniques (Bordes et al., 2013) account for asymmetry to some extent, TARE (Embedding knowledge graphs based on Transitivity and Asymmetry of Rules) (Wang et al., 2018) incorporates transitivity and asymmetry of relations in the vector representations by utilizing non-negative matrix factorization technique. This model captures the ordering of relations and infers potential new relations based on the ordering of existing relations, as well as the properties of asymmetry and transitivity of rules.

### Factorization-based embedding methods

Tensor factorization methods have been widely applied to various problems in machine learning and data mining. Tensors encode multi-dimensional data and can represent multi-relational data naturally. Such tensors are then factorized to obtain latent representations for the entities and their relationships. Tensor factorizations are an extended form of matrix factorization or decomposition that can be applied to account for the existence of relations in knowledge graphs. A knowledge graph can be described as a third-order binary tensor; each element corresponds to a triple (subject, relation, object). The entries of the tensor are either one indicating a fact (i.e., true relation between two entities) or zero otherwise (i.e., either negative or missing relation), and the relation interactions in the knowledge graph are relation-specific matrices. According to Nickel, Tresp & Kriegel (2011), the primary motivations behind applying tensor factorization for relational learning are, (1) it provides modeling simplicity when representing relation as a three-way tensor of higher orders, and (2) tensor methods require no prior knowledge about the structure or the independent variables. Furthermore, tensor factorization is considered a suitable alternative to the Markov Logic Networks that requires the structure to be known. Also, factorization methods perform well, despite the high-dimensional data and sparse problems commonly faced in relational learning. Traditional and well-known tensor factorization approaches such as (Tucker, 1966; Harshman & Lundy, 1994; Harshman, 1978) have limited scalability to large knowledge graphs and cannot perform collective learning (Nickel, Tresp & Kriegel, 2011). On the other hand, RESCAL is capable of collective learning. In other words, it is a tensor-based relational learning approach that models the pairwise interactions between the subject and object entities and the relations (i.e., three-way model), in multi-relational data. It first performs rank-*r* factorization, where each slice of the tensor represents a relation-specific matrix *χ*_*p*_ that is factorized as follows:

(10) $${\chi _p} \approx {\bf A}{{\bf M}_{\bf p}}{{\bf A}^{\rm T}},\ {\rm for}\ p = 1, \ldots,m$$

where **A_n__×__d_** is a matrix latent factor representation of *n* entities of *d* feature dimensions. **M_p_** is an asymmetric relation-specific matrix containing the interactions of the latent factor in the *p*-th relation. It then minimizes the function that captures the latent semantics for each entity appearing as *subject* or *object* within a triple:

(11) $$\mathcal{L} = \displaystyle{1 \over 2}\sum\limits_p {[{\chi _p} - {\bf s}{{\bf M}_{\bf p}}{{\bf o}^{\rm T}}]^2}$$

The above formulation preserves the asymmetry between entities and whether they occur as subjects (Nickel, Tresp & Kriegel, 2011). This property is essential for modeling certain types of relations that may exist in biological datasets and ontologies; for example, *subClassOf* and *instanceOf* relations. One of the main limitations of RESCAL is that it is inefficient to train as it has quadratic run-time and scales poorly to large knowledge graphs. These limitations of RESCAL led to the development of other KG embedding methods, DistMult (Yang et al., 2014), Holographic Embeddings (HolE) (Nickel, Rosasco & Poggio, 2016) and ComplEx (Trouillon et al., 2016). DistMult avoids the computational complexity of RESCAL by diagonalizing the relation-specific matrix, thereby restricting its modeling capability to symmetric relations only. However, DistMult suffers from other limitations because its relations are represented by diagonal matrices, which causes difficulty with longer rules extraction. These rules require modeling of more complex relation semantics. To avoid all previous limitations, Nickel, Rosasco & Poggio (2016) then proposed HolE. HolE exploits the expressive of RESCAL with the efficiency and simplicity of DistMult. It uses a circular correlation operator to construct a composite feature representation of the *subject* and *object*, which is then semantically matched (i.e., dot product) with the relation vector to score the fact. This approach offers expressiveness to model asymmetric relation while maintaining low computational complexity. ComplEx (Trouillon et al., 2016), on the other hand, explores the use of complex numbers to model several relational patterns such as symmetry and anti-symmetry. TriModel (Mohamed & Nováček, 2019) combines DistMult and ComplEx methodologies to investigate the possibility of encoding symmetric and asymmetric relations.

SimplE (Kazemi & Poole, 2018), a recent powerful factorization technique learns interpretable and expressive embeddings, allowing specific background knowledge to be encoded through weight sharing. It addresses the issue of independence between subject and object entities through the inverse relation as a way to capture the dependance between entities and exploit the similarity and dissimilarity information as they occur in different roles (i.e., as subjects and objects in a relation). As a result, SimplE similarity function is defined as:

(12) $$score({\bf s},{\bf o}) = \displaystyle{1 \over 2}(\langle {{\bf s}_{{e_i}}},{\bf p},{{\bf o}_{{e_j}}}\rangle + \langle {{\bf s}_{{e_j}}},{{\bf p}^{ - 1}},{{\bf o}_{{e_i}}}\rangle )$$

The SimplE model authors (Kazemi & Poole, 2018) defined $\langle {\bf s},{\bf p},{\bf o}\rangle$ to be $\langle {\bf s},{\bf p},{\bf o}\rangle = ({\bf s}{\rm \odot }{\bf p}) \cdot {\bf o}$ where $\odot$ represents the element-wise (Hadamard) product and the :amp:odot; represents the dot product.

### Graph convolutional networks-based embedding methods

Recently, graph convolutional networks (GCNs) have also been utilized to learn KG embeddings for several tasks, including link prediction and entity classification. Unlike knowledge graph embedding methods mentioned above, which mainly employ shallow models, GCNs have emphasized their performance using deep models features learning. Relational Graph Convolutional Network (R-GCN) (Schlichtkrull et al., 2018) is the first method to show that GCNs can be applied to model knowledge graphs data (i.e., relational data). R-GCN has an encoder-decoder framework that consists of two parts: first, the encoder model which learns the latent vector representations of entities and their interactions, and second, a decoder model which could employ any matrix factorization technique (DistMult (Yang et al., 2014)). R-GCN revealed shallow factorization models, such as DistMult, can be significantly improved by learning the encoder through deep, multi-layer inference known as message passing (described in Duvenaud et al. (2015) and Kipf & Welling (2016)), the decoder model computes the score similar to DistMult model (Yang et al., 2014) as follows:

(13) $$score({\bf s},{\bf o}) = {{\bf s}^{\rm T}}{{\bf R}_p}{\bf o}$$

where **R** is a diagonal relation-specific associated with the *p*−th relation.

Furthermore, ConvE (Dettmers et al., 2018); a multilayer GCN is another model which learns parameter-efficient vector representations which attempt to present a tradeoff between the lack of expressiveness of shallow models and the scaling and overfitting problems of deep models. ConvE model forms a 2-D matrix from the *subject* and the *predicate* vector representations, which are used as inputs to the convolutional layers. The resulting feature maps tensor is then reshaped into a vector through *vec*() and projected into the lower dimension by **W** and matched by the *object* vector representation through the dot product which computes its interaction and generate the corresponding as follows.

(14) $$score({\bf s},{\bf o}) = f(vec(f(concat({\bf S},{\bf P})*\omega )){\bf W}){\bf o}$$

where *f*() is the rectified non-linear unit (Nair & Hinton, 2010) activation functions, *concat*(*S*, *P*) is 2-D matrix concatenation of the ${\bf s}$ and ${\bf p}$ embedding vectors, *ω* is the 2-D convolutional filters and **W** is a linear transformation projection matrix. Similar to ConvE, ConvKG (Nguyen et al., 2018) forms a 2-D matrix which instead consists of all the 3 triple elements: *subject*, *predicate* and *object* vector representations. This is used that as input to the convolutions layers which applies 1-D filters. The resulting features maps are concatenated into a single vector and matched with the weights vector ${\bf w}$ through a dot product to generate the score. Therefore the resulting scoring function is as follows:

(15) $$f(f(concat({\bf s},{\bf p},{\bf o})*{\rm \omega} )w$$

where *f*() is the rectified non-linear unit (Nair & Hinton, 2010) activation functions. *ω* is the 1-D convolutional filters. ConvKB is said to model global relationships between same dimensional entities unlike ConvE (Nguyen et al., 2018). Lastly, Weighted GCN (Shang et al., 2019) utilizes learnable relational specific scalar weights, while Composition-based GCN (COMPGCN) (Vashishth et al., 2019) is a recent method developed by systematically leveraging different composition operators introduced in various knowledge embedding methods.

### Multimodal embedding methods

While the methods discussed above primarily consider the rich structure of the knowledge graphs, other methods can achieve improved performance by exploiting different types of data representations such as extracting text-based or image-based features. The combining of features from two or more modes of representation has been incorporated in various applications to improve classification or clustering tasks such as better word similarity (Collell, Zhang & Moens, 2017). One of the first KG methods to show multi-modal feature integration with the textual content is Neural Tensor Network (Socher et al., 2013), wherein pre-trained word vectors initialize the mode. Subsequently Wang et al. (2014) showed the effective utilization of both structured and unstructured information by aligning the KG corpus with the text corpus in a joint model and defining an aggregated loss for both representations. In similar work that extends TransE, Description-embodied knowledge graph (DKRL) (Xie et al., 2016a) utilizes the textual descriptions of KG entities, and define two vector representations for each entity: one that encodes the structural information and another that captures the textual description features. On the other hand, Image-embodied Knowledge Representation Learning (IKRL) (Xie et al., 2016b) modified the loss function introduced in TransE to account for both the structural and visual features of the entities in the knowledge graph (image features). Combining all three modes of representation associated with knowledge graph entities has also been studied in (Sergieh et al., 2018). These include: the translational structural features, the Image-embodied Knowledge Representation Learning (IKRL) which encodes image-based features, and the Description-Embodied Knowledge Representation Learning (DKRL) which encodes text-based features were incorporated into a single model. The experiments demonstrated that integrating multi-modal performance is better than the best IKRL model, and the other single models. Additionally, some models exploit the metadata as a source of prior knowledge of knowledge graph entities. Such models learn from the information available such as relation or entity types, which could improve inference about missing triples (Lv et al., 2019; Wang et al., 2019a; Chen et al., 2019).

Furthermore, multi-source Knowledge Representation Learning model (Tang et al., 2019) is multimodal in terms of combining features and combining models. It combines entity descriptions, hierarchical types, and textual relations with triple facts. Specifically, for entity descriptions, it uses convolutional neural networks to generate the representations. The hierarchical type computes the projection matrices of entities to the hierarchical types they belong to using weighted hierarchy encoders. For text-based feature generation, it uses a sentence-level attention mechanism.

## Evaluation and Comparison Data

### Data sources

Table 1 shows knowledge graph sources centered around biological entities such as genes, diseases, and drugs that were developed through the accumulation of several databases and adding bio-ontologies such as Gene Ontology (GO) (Ashburner et al., 2000), Disease Ontology (DO) (Schriml et al., 2012) and Human Phenotype Ontology (HPO) (Robinson et al., 2008). As a source of background knowledge, we utilized a knowledge graph constructed by Alshahrani et al. (2017a) centered around biological entities and their interrelations and is complemented with domain-specific biomedical ontologies. The graph consists of 29,984 curated disease-genes relations from DisGeNet (Piñero et al., 2015), 432,512 drug-target relations from STITCH (Kuhn et al., 2012), and 240,775 Human protein interactions from STRING (Szklarczyk et al., 2015) filtered by the confidence score selected above 700. We also include 6,190 drug–indications relations and 81,006 drug-side effects from SIDER (Kuhn et al., 2010). The graph also contains 244,105 Human GO annotations from SwissProt The UniProt Consortium Huntley et al. (2015), 153,575 gene-phenotype, and 84,508 disease-phenotype annotations (Köhler et al., 2014; Hoehndorf, Schofield & Gkoutos, 2015a). Table 1 in Appendix D provides additional statistics of relations and entities. We normalized and mapped all database identifiers to their ontology identifiers, as described in Alshahrani et al. (2017a). This knowledge graph has also been utilized in several studies and was used for benchmark analysis, as indicated in (Agibetov & Samwald, 2018a; Liu et al., 2018; Agibetov & Samwald, 2018b).

**Table 1:** The biological knowledge graph sources.

Relation	Relation database source	Source type	Target type
has function	Uniprot	Gene (Entrez)	Function (Gene Ontology)
has disease annotation	DisGeNet	Gene (Entrez)	Disease (Disease Ontology)
has interaction	STRING	Gene (Entrez)	Gene (Entrez)
has sideeffect	SIDER	Drug (PubChem)	Phenotype (Human phenotype)
has indication	SIDER	Drug (PubChem)	Disease (Disease Ontology)
has target	STITCH	Gene (Entrez)	Drug (PubChem)
has gene phenotype	HPO annotations	Gene (Entrez)	Phenotype (Human Phenotype Ontology)
has disease phenotype	Hoehndorf, Schofield & Gkoutos (2015b)	Disease (Disease ontology)	Phenotype (Human Phenotype Ontology)

We also included Hetionet (Himmelstein et al., 2017) that contains several types of biological entities and relations. Specifically, we extracted a subset of Hetionet that consists of the following relations: *treats, presents, associates* and *causes* with a 155,679 total number of relation edges. Table 2 shows a summary of the statistics of relations and entities.

**Table 2:** Number of relation edges and participating entities in each relation of subset of Hetionet dataset. The semantic of each relation is as follows: treats relation: compound–treats–disease; presents relation: disease–presents–symptom; associates relation: disease–associates–gene; causes relation: compound–causes–sideeffect.

Relation	# of relation edges	# of source entities	# of destination entities
treats relation	755	387 (Drugbank)	77 (Disease ontology)
presents relation	3,357	133 (Disease ontology)	415 (MeSH)
associates relation	12,623	134 (Disease ontology)	5,392 (Entrez Gene)
causes relation	138,944	1,071 (Drugbank)	5,701 (SIDER)

### Evaluations metrics

To solve biology-related problems, using an in silico approach, requires several evaluation metrics. Here, we describe several evaluation metrics, including the hits@10 (%) and the mean rank metric, as they are accepted performance metrics for knowledge graph embedding methods. We also include the Area Under the Receiver Operating Characteristic Curve (AUROC) and the F-score.

- Hits@10
  hits@10(%) is one of the performance metrics used to evaluate the results of embedding method ${\rm \dot H}$ere we report the proportions of the correctly predicted entities ranked in the top 10 among all entities of the same type, for all tested triples.
- Mean rank
  Another metric for reporting the predictions in knowledge graph methods is mean rank. Here, we followed a similar procedure as in Bordes et al. (2011). For each tested entity, we applied the model by fixing the first part, which corresponds to the subject and enumerating all of the objects of the same entity type. We sorted the models’ scores in descending order to obtain the rank of the correct object and reported the mean of all ranks in the test triples.
- Area under the Receiver Operating Characteristic Curve (AUROC/AUC)
  Area under the Receiver Operating Characteristic Curve (Fawcett, 2006) is a useful evaluation metric for binary classifiers. To compute AUC, the true positive rate (TPR, also known as sensitivity or recall), and the false-positive rate (FPR) must be calculated for each threshold. The FPR and the TPR constitute the *x*-axis and the *y*-axis of the ROC curve, respectively. The TPR is the proportion of correctly predicted positive samples over the total number of positive samples defined as $TPR = \textstyle{{TP} \over {TP + FN}}$, while the FPR is the proportion of incorrectly predicted negatives over the total number of negative samples defined as $FPR = \textstyle{{FP} \over {FP + TN}}$. Area under the Receiver Operating Characteristic Curve can be applied in different forms, to predict the existence of relations in the binary form. We applied macro-AUC, the neural networks model scores as the thresholds. At a given threshold, TP is the number of pairs predicted as associated, and their true label retrieved from the database is positive (i.e., the number of associated disease-genes as recorded in DisGeNet). At the same time, the TN is the number of pairs predicted as non-associated, and there is no record in the database of this association (i.e., when the model correctly predicted the negative class). We also applied micro-AUC, where the classifiers’ ranks are considered the thresholds. In this case, we consider as TP, all pairs ranked above this threshold and are found in the respective database as associated, while we treat all non-associated pairs as negatives. The micro-AUC can also be interpreted as computing the AUC in the multiclass setup. For example, in the disease-gene prediction, we treat each disease as a different class. Therefore, at each rank, we compute the TPR and FPR by aggregating all disease classes’ predictions globally.
  We computed the micro-AUC (results are shown in Appendix A) to examine the effects of a ranking scheme on the AUC results as the absence of “rue negatives” remains an issue in the evaluation of biological results due to the possible incompleteness inherent in the curated databases. Therefore, we are not only interested in evaluating whether a relation/edge exists (as in the macro-AUC), but also in its rank among other possible associations which is relevant and useful in prioritization tasks typical of biomedical applications. The reason is that the appropriate ranking of genes associated with a disease or targets and their associated drugs can make the experimental validation process fast and less expensive.
- F-Score
  The F1-Score is the harmonic mean of precision and recall. It is defined as follows:
  (16) $$F1 - score = 2*\displaystyle{{recall \times precision} \over {recall + precision}}$$
  where the recall is the same as the TPR *recall* = *TPR* defined above, and the precision is defined as $precision = \textstyle{{TP} \over {TP + FP}}$, which is the proportion of true positives over the number of predicted positives (also known as the positive predictive value (PPV)). The F1-score is known to give more realistic measures due to its insensitivity to class imbalance (Fawcett, 2006). The results of this evaluation metric are shown in Appendix A.

### Training and evaluation

To train the models, we used a positive set consisting of the true associations for each relation, while the negative set is constructed by sampling an equal number of negative associations from the pool of unknown associations. We strictly require the negative associations to be between entities of the same types. For example, for the *has–disease–annotation* relation, the positive set is the true associations (as in DisGeNet curated gene-disease associations) between disease and gene entities in the knowledge graph, while the negative set is between the set of genes and diseases that are not associated. To maintain fair comparisons, we fixed the training and testing triples in all of our experiments across different methods. We also fixed the neural network architecture to ensure one hidden layer is double the size of the input features. We ran all the methods using the default parameters described by the original authors, except with RESCAL, as the original implementation of RESCAL suffers from scalability issues. Other knowledge graph-based methods circumvent this limitation by setting the regularization parameter to zero when comparing with RESCAL as a baseline (Bordes et al., 2013; Wang et al., 2014). We found that although this could allow us to run the method, it results in drastically lower performance than when fixing this parameter to a reasonable value such as 0.01. As a result, we used OpenKE for TransE, Rescal and SimplE (Han et al., 2018), which also uses C++ based implementation for some underlying functions such as negative sampling. This functionality is essential for large knowledge graphs, such as our knowledge graph. For the Poincaré method, we used Gensim implementation For the Poincaré method, we used Gensim implementation (Řehůřek & Sojka, 2010). For R-GCN, we used Deep Graph Library (DGL) (Wang et al., 2019b). Additionally, we have conducted experiments to show the effect of different parameter settings on each method’s output (Appendix C). For this reason, we identified the number of dimensions (*dim*), learning rate (*lr*), the number of epochs, and the number of minibatch which are standard parameters among all of the knowledge graphs methods and are known to be used for parameter optimization (Bordes et al., 2013; Lin et al., 2015b; Wang et al., 2014).

## Results

### Relation prediction

**Figure 1:**
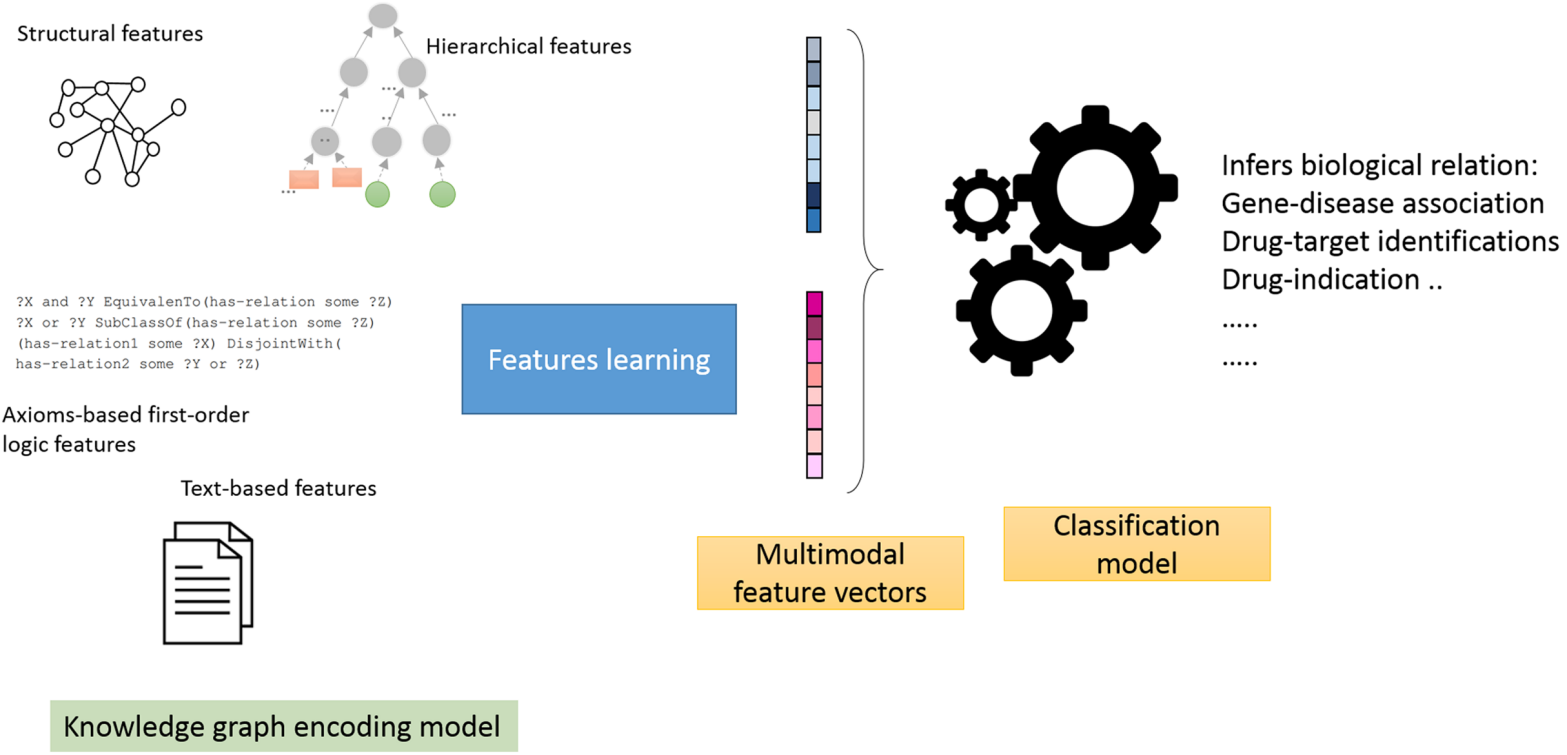
Illustration of the general workflow of our experiments.

Using the knowledge graph described in (refer to “Data Sources” section), we conducted a comparative experimental analysis between six knowledge graph embedding methods: Walking RDF and The Web Ontology Language (OWL), TransE, Poincaré embeddings, RESCAL, SimplE and R-GCN. Each method belongs to one of the categories introduced in “Methods for Knowledge Graph Embedding”. We have specifically selected the most representative and successful method of each category for our comparison (Su et al., 2020; Yue et al., 2020; Holter et al., 2019). We trained each method as feature generation and end-to-end models:

- Feature generators models: in this mode, we employ a two-stage pipeline (Fig. 1), which consists of treating knowledge graphs as feature generators followed by a link prediction model (Alshahrani et al., 2017a). The aim is to assess how well the generated features predict biological relations. Briefly, we selected neural networks as the link prediction model, given its capability to learn non-linear functions and reveal intricate graph patterns encoded in pairs of feature vectors (Bishop, 2006). We then used the neural model scores produced by the sigmoid function in the last layer to predict the object entity in the test triple.
- End-to-end models: this corresponds to the native mode in which knowledge graph embedding methods are trained. We trained each method and applied the model on the test triples. Briefly, we use the learned feature vectors or matrices (i.e., in case of RESCAL and R-GCN) to compute the scoring functions. For example, in TransE, we compute the *L*1 norm defined below and compare the scores of each subject in the test triples to all of the objects (which excludes the object entities in the training set). The results for this section are shown in Appendix B.
  (17) $$score({\bf s},{\bf o}) = d({\bf s} + {\bf p},{\bf o})$$
  For RESCAL, we computed the scores as defined by the loss function introduced in Nickel, Tresp & Kriegel (2011) as follows:
  (18) $$score({\bf s},{\bf o}) = {\bf s}{{\bf M}_{\bf p}}{{\bf o}^{\rm T}}$$
  While in the Poincaré method, they defined the distance in the hyperbolic space as follows:
  (19) $$score({\bf s},{\bf o}) = arcosh(1 + 2\displaystyle{{||{\bf s} - {\bf o}{{||}^2}} \over {{{(1 - ||{\bf s}||)}^2}{{(1 - ||{\bf o}||)}^2}}})$$
  Where *arcosh* is the inverse hyperbolic cosine and ||.|| is the *L*2−*norm*.
  The SimplE embedding model (Kazemi & Poole, 2018) scoring function is defined as follows:
  (20) $$score({\bf s},{\bf o}) = \displaystyle{1 \over 2}(\langle {{\bf s}_{{e_i}}},{\bf p},{{\bf o}_{{e_j}}}\rangle + \langle {{\bf s}_{{e_j}}},{{\bf p}^{ - 1}},{{\bf o}_{{e_i}}}\rangle )$$
  Lastly, the Relational Graph Convolutional Networks (R-GCN) (Schlichtkrull et al., 2018) model scoring function is defined as mentioned above:
  (21) $$score({\bf s},{\bf o}) = {{\bf s}^{\rm T}}{{\bf R}_p}{\bf o}$$
  Where **R** is a diagonal relation-specific associated with the *p*−th relation.
  We also designed our experiments to determine how partial and free settings could affect the study results.
- Partial: when using this setting, we removed 20% of the relations links for each biological relation (i.e., for testing) but retained 80% of these links to generate the features for each method (i.e., for training). After that, we trained the neural network model on each pair of entities in the relation or applied the knowledge graph embedding method in the native mode and computed the scoring functions in the end-to-end models. Learning from known associations when predicting future or possible links resembles a widely used principle (known as guilt-by-association) for evaluating computational predictions in biomedical applications (Pahikkala et al., 2014; Thafar et al., 2019; Alshahrani et al., 2017b).
- Free: when using this setting, all relations links were removed to generate the features for the nodes in the relations. As our knowledge graph is heterogeneous and multi-relational, the nodes retain connections via other relations. Compared to the previous simplified setting, this resembles a more realistic but challenging approach and could provide a more reliable and robust evaluation scheme for practical application.

The hit@10 in Table 3 shows Walking RDF and OWL outperforms other methods in four and five relations out of a total of eight relations in the *partial* and *free* settings, respectively. TransE and SimplE followed by showing overall better results than other methods in both the settings. The performance degradation between *partial* and *free* is clear as the latter doesn’t depend on any known associations in the tested relation edges, but purely on other the entities and relation features connected within the knowledge graph. The hit@10 results on the subset of Hetionet knowledge graph are in Table 4 and shown to perform similar performance indicating the advantage of random walk and R-GCN-based methods. The Mean rank results show similar patterns and are included in “Appendix B”.

**Table 3:** The top hit@10 results (*partial* and *free* settings) for relation prediction of our biological knowledge graph as feature generators models. *Partial* and *free* settings are two evaluation strategies correspond to: partially removing 80% of the relation edges and completely removing all relation edges, respectively.

Relation	Walking RDF/OWL	TransE	Poincaré	Rescal	SimplE	R-GCN
	*partial*					
has function	12.97	10.51	7.36	5.62	**19.20**	9.35
has interaction	11.51	**13.18**	3.75	8.62	8.60	11.40
has disease annotation	**31.10**	26.31	21.76	19.40	24.02	20.42
has sideeffect	22.42	**25.70**	5.70	17.07	17.26	17.21
has indication	**26.09**	18.40	9.77	13.09	14.66	11.83
has target	15.32	13.70	7.68	5.50	**20.28**	10.93
has gene phenotype	**15.23**	14.89	8.67	8.59	8.97	10.07
has disease phenotype	**34.69**	19.86	11.36	10.32	8.61	7.22
	*free*					
has function	**3.16**	2.54	0.43	0.75	2.31	2.56
has interaction	**4.14**	2.53	1.84	0.74	2.81	0.89
has disease annotation	**22.51**	21.90	8.02	14.24	10.96	15.75
has sideeffect	18.14	**21.20**	0.05	13.94	5.82	6.77
has indication	**14.78**	11.95	5.25	9.61	9.09	13.40
has target	0.24	0.26	0.06	0.12	**1.73**	0.63
has gene phenotype	9.08	**9.61**	0.94	6.80	1.94	4.65
has disease phenotype	**10.83**	4.73	1.10	3.05	1.59	1.89

**Note:**

The highest performing method is indicated in bold.

**Table 4:** The top hit@10 results for relation prediction on the subset of Hetionet dataset as feature generators models.

Relation	Walking RDF/OWL	TransE	Poincaré	Rescal	SimplE	R-GCN
treats relation	53.79	55.62	22.06	47.01	40.39	**67.54**
presents relation	**31.28**	24.85	13.26	26.93	14.43	21.13
associates relation	**8.32**	6.53	3.71	4.59	0.95	2.61
causes relation	**16.78**	13.29	8.71	13.83	15.68	8.43

**Note:**

The highest performing method is indicated in bold.

### Clustering and visualization

**Figure 2:**
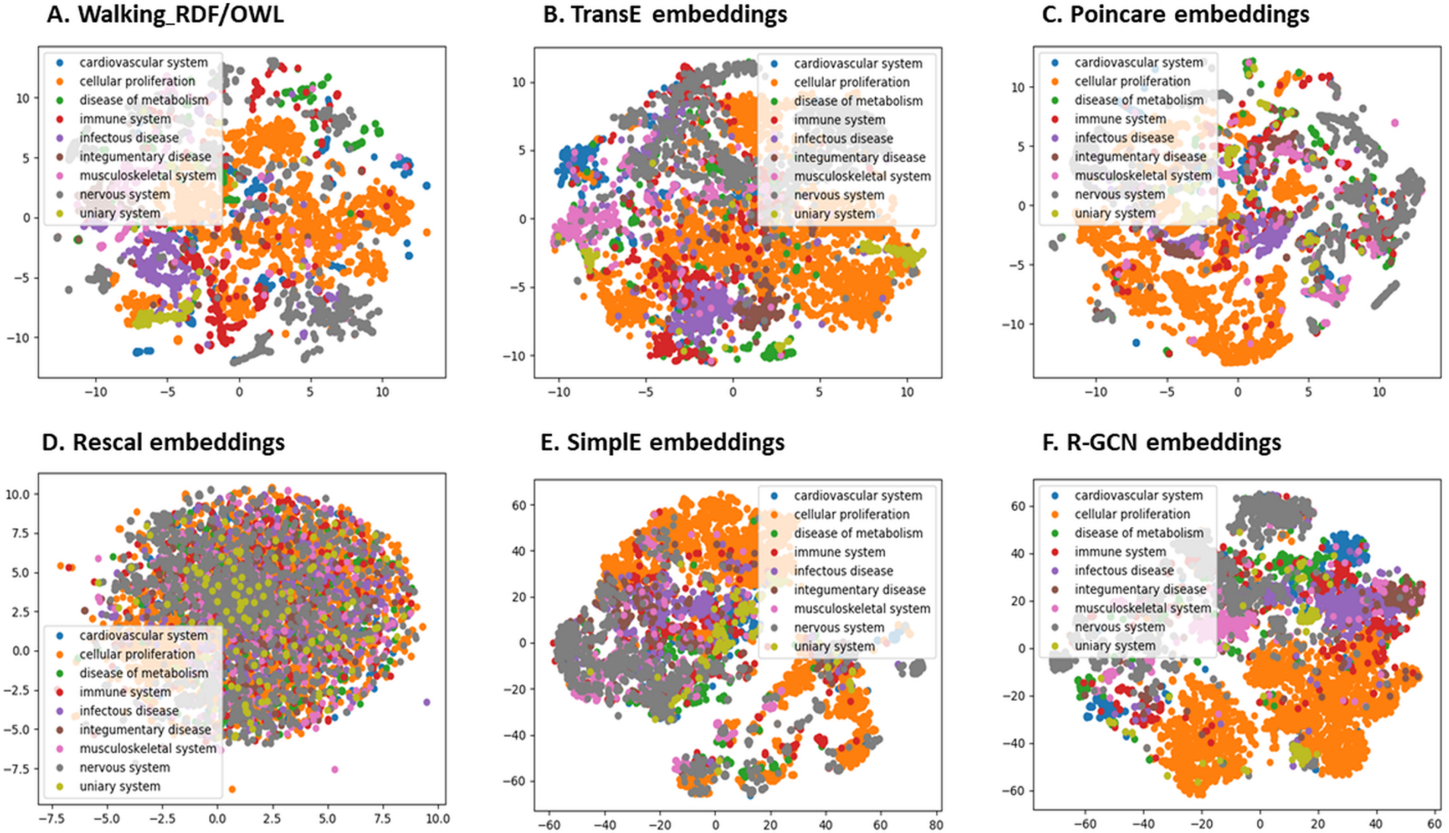
The 2-D t-SNE plot of Disease ontology top categories according to each embedding method. (A) Walking RDF/OWL, (B) TransE embeddings, (C) poincare embeddings, (D) rescal embeddings, (E) simple embeddings, (F) R-GCN embeddings.

We further evaluated the quality of the features produced by each method and assessed how much information they preserved when embeddings vectors are projected into two-dimensional space. We used a recent technique called t-Distributed Stochastic Neighbor Embedding (t-SNE) (Maaten & Hinton, 2008). This method can reveal the local and global features encoded by the feature vectors and thus can be used to visualize clusters within the data (Maaten & Hinton, 2008). We applied t-SNE to 6,353 feature vectors, which refer to disease nodes from the knowledge graph. We aim to identify clusters of Disease Ontology (DO) upper classes representing disease classes of similar phenotypes and mechanisms (Schriml et al., 2011). The t-SNE plot is shown in Fig. 2, the disease data points can be grouped into nine clusters with varying degrees of separability and overlap. The methods Walking RDF and OWL, Poincaré, SimplE, and R-GCN showed more distinct groups than RESCAL, which shows no clusters. The results illustrate how the features of each method capture the original structure and reflect the similarity between the data points in the knowledge graph. Moreover, we used Kmeans clustering to evaluate and quantify each method’s vector representations with and without t-SNE, as shown in Table 5. To estimate the cluster quality for each embedding method, we have used the purity and normalized mutual information (NMI) metrics defined below:

(22) $$purity(Y,C) = 1/N\sum\limits_k max|{c_k} \cap {y_j}|$$

(23) $$NMI(Y,C) = \displaystyle{{2 \times I(Y;C)} \over {[H(Y) + H(C)]}}$$

where *Y* = {*y*_1_,*y*_2_,..,*y*_*j*_} is the class labels, *C* = {*c*_1_,*c*_2_,..,*c*_*k*_} is the cluster labels, *I*(*Y*;*C*) is the mutual information and *H*(*C*) is the entropy.

**Table 5:** Disease categories clusters analysis between different knowledge graph methods. Purity and normalized mutual information (NMI) are used to measure clusters’ goodness.

Relation	without t-SNE		with t-SNE	
	Purity	NMI	Purity	NMI
Walking RDF/OWL	**0.682**	**0.438**	0.507	**0.237**
TransE	0.540	0.245	0.565	0.254
PoincarÃ©	0.660	0.367	**0.569**	0.222
Rescal	0.510	0.165	0.434	0.032
SimplE	0.562	0.237	0.556	0.227
R-GCN	0.563	0.226	0.557	0.223

**Note:**

The highest performing method is indicated in bold while the second highest is underlined.

Table 5 shows that Poincaré performs better in this task than in the relations prediction, which could be attributed to its ability to model hierarchical features, as discussed in “Methods for Knowledge Graph Embedding”.

## Limitations and Future Work

Despite the promising results, there are several issues regarding training and evaluation in the biological and biomedical domains. The likely incompleteness of biological knowledgebases and the principle of Open World Assumption (OWA) in the knowledge graphs and Linked Data, which states that non-observed facts are not necessarily false, should guide the choice of the objective function. Our experiments showed that the distance-based method TransE and Walking RDF/OWL generally performed better than other methods, likely attributed to the way the loss is defined. For example, in TransE, the pairwise ranking loss is defined as below:

(24) $$\mathcal{L} = \sum\limits_{(s,p,o) \in \mathcal{L} \sum\limits_{({s}^{\prime},p,o) \in \mathcal{L}^{\prime}}} [{\rm{\gamma}} + d({\bf s} + {\bf p},{\bf o}) - d({\bf {s}^{\prime}} + {\bf p},{\bf o})]$$

where $\mathcal{L}$ is the positive triples and $\mathcal{L}^{\prime}$ is the negative triples, $\mathcal{L}^{\prime} = ({s}^{\prime},p,o) \cup (s,p,{o}^{\prime})$.

This loss favors low values of the scoring function for positive samples $\mathcal{L}$ than those of the negative samples $\mathcal{L}^{\prime}$. Such ranking loss formulation is essential as it does not assume that the negative samples are necessarily false, but they are less likely than the positive samples, which is the case in biological databases, in which there are no true negatives. Similarly, Walking RDF/OWL as a feature generation method based on the Skip-gram model (Mikolov et al., 2013)

(25) $$J = 1/N\sum\limits_{t = 1}^N \sum\limits_{ - c \le j \le c,j \ne } logp({w_{t + j}}|{w_t})$$

where *c* is the size of the context window *c*. Skip-gram optimizes the probability knowledge graph entities co-occur within the context of the same relations. Entities that constitute the positive triples should have more similar vector representations than entities that make up negative triples. Although this ranking constraint is not enforced in the skip-gram formulation above, as in TransE, it would be interesting to incorporate such property into random walk-based methods.

Here we have primarily shown the opportunities of applying knowledge graphs as a prediction tool in the framework of relation/link prediction. However, employing knowledge graph embedding to find incomplete or flagging inaccurate information stored in biomedical warehouses not only serves as a direct application of knowledge graph embedding methods (Nickel et al., 2015; Wang et al., 2017) but would also solve a critical problem in the biomedical domain. Knowledge graph embedding methods employ various methodological techniques and model different relational patterns and properties of the knowledge graphs and scale differently with the knowledge graphs’ size. However, their actual collective performances on large biomedical datasets do not always prefer one method or a certain category of methods over the others. Thus, choosing the appropriate method remains complex and data-dependent.

One of the significant limitations of knowledge graph embedding methods is that they handle the qualitative type of information; this restricts its use to consider only the edge semantic. Incorporating the edge weight would effectively increase its application to quantitative information, which widely characterize biological data and its interactions (AlShahrani, 2019).

Another potential application and currently active research topic is using zero-shot learning (i.e., learning to predict new classes with zero instances in the training phase). With the knowledge graph, this could be made possible as knowledge graphs are a natural fit for multimodal data sources directly (refer to the Multimodal embedding methods section), This is mainly attributed to the use of Semantic Web and Linked Data tools for identification and sharing in the biomedical domain (Candan, Liu & Suvarna, 2001; Callahan et al., 2013).

## Conclusion

Knowledge graph representation learning is emerging as a new and interesting paradigm for learning and prediction. In this work, we provide an overview of the main categories of knowledge graphs embedding methods and briefly describe them using RDF and Linked data terminologies (AlShahrani, 2019). Then, we present the first large-scale evaluation of knowledge graph embedding methods in the biomedical domain; almost all results from knowledge graph embedding methods are based on two general-purpose datasets (i.e., FreeBase (Bollacker et al., 2008) and WordNet (Miller, 1995)). We show several potential applications and opportunities for utilizing representation learning of knowledge graphs for data analytics and prediction tasks in the biomedical field. Specifically, we distinguish our analysis by providing different realistic and commonly used strategies of training and evaluation used in the biomedical domains, but rarely compared against each other systematically and collectively (i.e., partial and free for several relations denoting several biomedical applications). We also demonstrate the effects on the performance results by treating knowledge graphs models in various modes and computing similarities under unified experimental setup with regard to widely used evaluation metrics in both the knowledge graphs and biomedical literature.

## Supplemental Information

10.7717/peerj-cs.341/supp-1 Supplemental Information 1 Appendix A.

10.7717/peerj-cs.341/supp-2 Supplemental Information 2 Appendix B.

10.7717/peerj-cs.341/supp-3 Supplemental Information 3 Appendix C.

10.7717/peerj-cs.341/supp-4 Supplemental Information 4 Appendix D.

## Additional Information and Declarations

### Data Availability

The following information was supplied regarding data availability: The analysis code, scripts with instructions and README are available at GitHub: https://github.com/monaalsh/kg-embeddings-in-biomedicine. Files necessary to generate the embedding are available at Figshare: Alshahrani, Mona (2020): Application and evaluation of knowledge graph embeddings in biomedical data. figshare. Dataset. DOI 10.6084/m9.figshare.13235195.v1.

## References

[ref-1] Agibetov A, Samwald M (2018a). Fast and scalable learning of neuro-symbolic representations of biomedical knowledge. https://arxiv.org/abs/1804.11105.

[ref-2] Agibetov A, Samwald M (2018b). Global and local evaluation of link prediction tasks with neural embeddings. https://arxiv.org/abs/1807.10511.

[ref-3] AlShahrani M (2019). Knowledge graph representation learning: approaches and applications in biomedicine.

[ref-4] Alshahrani M, Hoehndorf R (2018a). Drug repurposing through joint learning on knowledge graphs and literature. Biorxiv.

[ref-5] Alshahrani M, Hoehndorf R (2018b). Semantic disease gene embeddings (smudge): phenotype-based disease gene prioritization without phenotypes. Bioinformatics.

[ref-6] Alshahrani M, Khan MA, Maddouri O, Kinjo AR, Queralt-Rosinach N, Hoehndorf R (2017a). Neuro-symbolic representation learning on biological knowledge graphs. Bioinformatics.

[ref-7] Alshahrani M, Soufan O, Magana-Mora A, Bajic VB (2017b). Dannp: an efficient artificial neural network pruning tool. PeerJ Computer Science.

[ref-8] Ashburner M, Ball CA, Blake JA, Botstein D, Butler H, Cherry MJ, Davis AP, Dolinski K, Dwight SS, Eppig JT, Harris MA, Hill DP, Tarver LI, Kasarskis A, Lewis S, Matese JC, Richardson JE, Ringwald M, Rubin GM, Sherlock G (2000). Gene ontology: tool for the unification of biology. Nature Genetics.

[ref-9] Auer S, Bizer C, Kobilarov G, Lehmann J, Cyganiak R, Ives Z, Aberer K (2007). Dbpedia: a nucleus for a web of open data. The Semantic Web.

[ref-10] Bishop CM (2006). Pattern recognition and machine learning (Information Science and Statistics).

[ref-11] Biswas S, Mitra P, Rao KS (2019). Relation prediction of co-morbid diseases using knowledge graph completion.

[ref-12] Bollacker K, Evans C, Paritosh P, Sturge T, Taylor J (2008). Freebase: a collaboratively created graph database for structuring human knowledge.

[ref-13] Bordes A, Usunier N, Garcia-Duran A, Weston J, Yakhnenko O (2013). Translating embeddings for modeling multi-relational data.

[ref-14] Bordes A, Weston J, Collobert R, Bengio Y (2011). Learning structured embeddings of knowledge bases.

[ref-15] Callahan A, Cruz-Toledo J, Ansell P, Dumontier M (2013). Bio2rdf release 2: improved coverage, interoperability and provenance of life science linked data.

[ref-16] Candan KS, Liu H, Suvarna R (2001). Resource description framework: metadata and its applications. ACM SIGKDD Explorations Newsletter.

[ref-17] Carlson A, Betteridge J, Kisiel B, Settles B, Hruschka ER, Mitchell TM (2010). Toward an architecture for never-ending language learning.

[ref-18] Chang K-W, Yih SW-T, Yang B, Meek C (2014). Typed tensor decomposition of knowledge bases for relation extraction.

[ref-19] Chen M, Zhang W, Zhang W, Chen Q, Chen H (2019). Meta relational learning for few-shot link prediction in knowledge graphs. https://arxiv.org/abs/1909.01515.

[ref-20] Collell G, Zhang T, Moens M-F (2017). Imagined visual representations as multimodal embeddings.

[ref-21] Davis R, Shrobe H, Szolovits P (1993). What is a knowledge representation?. AI Magazine.

[ref-22] Dettmers T, Minervini P, Stenetorp P, Riedel S (2018). Convolutional 2d knowledge graph embeddings. 32nd AAAI Conference on Artificial Intelligence, AAAI 2018.

[ref-23] Dong X, Gabrilovich E, Heitz G, Horn W, Lao N, Murphy K, Strohmann T, Sun S, Zhang W (2014). Knowledge vault: a web-scale approach to probabilistic knowledge fusion.

[ref-24] Duvenaud DK, Maclaurin D, Iparraguirre J, Bombarell R, Hirzel T, Aspuru-Guzik A, Adams RP (2015). Convolutional networks on graphs for learning molecular fingerprints.

[ref-25] Ebisu T, Ichise R (2017). Toruse: knowledge graph embedding on a lie group. https://arxiv.org/abs/1711.05435.

[ref-26] Ebisu T, Ichise R (2019). Generalized translation-based embedding of knowledge graph. IEEE Transactions on Knowledge and Data Engineering.

[ref-27] Ehrlinger L, Wöß W (2016). Towards a definition of knowledge graphs.

[ref-28] Färber M, Bartscherer F, Menne C, Rettinger A (2016). Linked data quality of DBpedia, Freebase, OpenCyc, Wikidata, and YAGO. Semantic Web.

[ref-29] Fawcett T (2006). An introduction to ROC analysis. Pattern Recognition Letters.

[ref-30] Gardner M, Mitchell T (2015). Efficient and expressive knowledge base completion using subgraph feature extraction.

[ref-31] Grover A, Leskovec J (2016). node2vec: scalable feature learning for networks.

[ref-32] Guo S, Wang Q, Wang L, Wang B, Guo L (2016). Jointly embedding knowledge graphs and logical rules.

[ref-33] Han X, Cao S, Lv X, Lin Y, Liu Z, Sun M, Li J (2018). Openke: an open toolkit for knowledge embedding.

[ref-34] Harshman RA (1978). Models for analysis of asymmetrical relationships among n objects or stimuli.

[ref-35] Harshman RA, Lundy ME (1994). Parafac: parallel factor analysis. Computational Statistics & Data Analysis.

[ref-36] Himmelstein DS, Lizee A, Hessler C, Brueggeman L, Chen SL, Hadley D, Green A, Khankhanian P, Baranzini SE (2017). Systematic integration of biomedical knowledge prioritizes drugs for repurposing. Elife.

[ref-37] Hoehndorf R, Schofield PN, Gkoutos GV (2015a). Analysis of the human diseasome using phenotype similarity between common, genetic, and infectious diseases. Scientific Reports.

[ref-38] Hoehndorf R, Schofield PN, Gkoutos GV (2015b). Analysis of the human diseasome using phenotype similarity between common, genetic and infectious diseases. Scientific Reports.

[ref-39] Holter OM, Myklebust EB, Chen J, Jimenez-Ruiz E (2019). Embedding owl ontologies with owl2vec. CEUR Workshop Proceedings.

[ref-106] Huntley RP, Sawford T, Mutowo-Meullenet P, Shypitsyna A, Bonilla C, Martin MJ, O’Donovan C (2015). The GOA database: gene ontology annotation updates for 2015. Nucleic Acids Research.

[ref-40] Kazemi SM, Poole D (2018). Simple embedding for link prediction in knowledge graphs.

[ref-41] Kipf TN, Welling M (2016). Semi-supervised classification with graph convolutional networks. https://arxiv.org/abs/1609.02907.

[ref-42] Köhler S, Doelken SC, Mungall CJ, Bauer S, Firth HV, Bailleul-Forestier I, Black GCM, Brown DL, Brudno M, Campbell J, FitzPatrick DR, Eppig JT, Jackson AP, Freson K, Girdea M, Helbig I, Hurst JA, Jähn J, Jackson LG, Kelly AM, Ledbetter DH, Mansour S, Martin CL, Moss C, Mumford A, Ouwehand WH, Park S-M, Riggs ER, Scott RH, Sisodiya S, Vooren SV, Wapner RJ, Wilkie AOM, Wright CF, Vulto-van Silfhout AT, Leeuw Nd, De Vries BBA, Washingthon NL, Smith CL, Westerfield M, Schofield P, Ruef BJ, Gkoutos GV, Haendel M, Smedley D, Lewis SE, Robinson PN (2014). The human phenotype ontology project: linking molecular biology and disease through phenotype data. Nucleic Acids Research.

[ref-43] Kuhn M, Campillos M, Letunic I, Jensen LJ, Bork P (2010). A side effect resource to capture phenotypic effects of drugs. Molecular Systems Biology.

[ref-44] Kuhn M, Szklarczyk D, Franceschini A, Von Mering C, Jensen LJ, Bork P (2012). STITCH 3: zooming in on protein-chemical interactions. Nucleic Acids Research.

[ref-45] Kulmanov M, Liu-Wei W, Yan Y, Hoehndorf R (2019). El embeddings: geometric construction of models for the description logic el++. https://arxiv.org/abs/1902.10499.

[ref-46] Lao N, Cohen WW (2010). Relational retrieval using a combination of path-constrained random walks. Machine Learning.

[ref-47] Lao N, Mitchell T, Cohen WW (2011). Random walk inference and learning in a large scale knowledge base.

[ref-48] Lehmann J, Isele R, Jakob M, Jentzsch A, Kontokostas D, Mendes PN, Hellmann S, Morsey M, Van Kleef P, Auer S, Bizer C (2015). Dbpedia-a large-scale, multilingual knowledge base extracted from wikipedia. Semantic Web.

[ref-49] Lin Y, Liu Z, Luan H, Sun M, Rao S, Liu S (2015a). Modeling relation paths for representation learning of knowledge bases. https://arxiv.org/abs/1506.00379.

[ref-50] Lin Y, Liu Z, Sun M, Liu Y, Zhu X (2015b). Learning entity and relation embeddings for knowledge graph completion. AAAI.

[ref-51] Liu Q, Wan R, Yang X, Zeng Y, Zhang H (2018). Generalized embedding model for knowledge graph mining. http://www.mlgworkshop.org/2018/papers/MLG2018_paper_5.pdf.

[ref-52] Lv X, Gu Y, Han X, Hou L, Li J, Liu Z (2019). Adapting meta knowledge graph information for multi-hop reasoning over few-shot relations. https://arxiv.org/abs/1908.11513.

[ref-53] Maaten Lv d, Hinton G (2008). Visualizing data using t-sne. Journal of Machine Learning Research.

[ref-54] Mikolov T, Sutskever I, Chen K, Corrado GS, Dean J (2013). Distributed representations of words and phrases and their compositionality.

[ref-55] Miller GA (1995). Wordnet: a lexical database for english. Communications of the ACM.

[ref-56] Mohamed SK, Nováček V (2019). Link prediction using multi part embeddings.

[ref-57] Mohamed SK, Nováček V, Nounu A (2020). Discovering protein drug targets using knowledge graph embeddings. Bioinformatics.

[ref-58] Nair V, Hinton GE (2010). Rectified linear units improve restricted boltzmann machines.

[ref-59] Nguyen DQ, Nguyen TD, Nguyen DQ, Phung D (2018). A novel embedding model for knowledge base completion based on convolutional neural network.

[ref-60] Nickel M, Kiela D (2017). Poincaré embeddings for learning hierarchical representations.

[ref-61] Nickel M, Murphy K, Tresp V, Gabrilovich E (2015). A review of relational machine learning for knowledge graphs. Proceedings of the IEEE.

[ref-62] Nickel M, Murphy K, Tresp V, Gabrilovich E (2016). A review of relational machine learning for knowledge graphs. Proceedings of the IEEE.

[ref-63] Nickel M, Rosasco L, Poggio T (2016). Holographic embeddings of knowledge graphs.

[ref-64] Nickel M, Tresp V, Kriegel H-P (2011). A three-way model for collective learning on multi-relational data. ICML.

[ref-65] Pahikkala T, Airola A, Pietilä S, Shakyawar S, Szwajda A, Tang J, Aittokallio T (2014). Toward more realistic drug-target interaction predictions. Briefings in Bioinformatics.

[ref-66] Perozzi B, Al-Rfou R, Skiena S (2014). Deepwalk: online learning of social representations.

[ref-67] Piñero J, Queralt-Rosinach N, Bravo A, Deu-Pons J, Bauer-Mehren A, Baron M, Sanz F, Furlong LI (2015). Disgenet: a discovery platform for the dynamical exploration of human diseases and their genes. Database.

[ref-68] Raedt LD, Kersting K, Natarajan S, Poole D (2016). Statistical relational artificial intelligence: logic, probability, and computation. Synthesis Lectures on Artificial Intelligence and Machine Learning.

[ref-69] Řehůřek R, Sojka P (2010). Software framework for topic modelling with large corpora.

[ref-70] Ribeiro LF, Saverese PH, Figueiredo DR (2017). struc2vec: learning node representations from structural identity.

[ref-71] Ristoski P, Paulheim H (2016). Rdf2vec: rdf graph embeddings for data mining.

[ref-72] Robinson PN, Köhler S, Bauer S, Seelow D, Horn D, Mundlos S (2008). The human phenotype ontology: a tool for annotating and analyzing human hereditary disease. American Journal of Human Genetics.

[ref-73] Schlichtkrull M, Kipf TN, Bloem P, Van Den Berg R, Titov I, Welling M (2018). Modeling relational data with graph convolutional networks.

[ref-74] Schlötterer J, Wehking M, Rizi FS, Granitzer M (2019). Investigating extensions to random walk based graph embedding.

[ref-75] Schriml LM, Arze C, Nadendla S, Chang Y-WW, Mazaitis M, Felix V, Feng G, Kibbe WA (2011). Disease ontology: a backbone for disease semantic integration. Nucleic Acids Research.

[ref-76] Schriml LM, Arze C, Nadendla S, Chang Y-WW, Mazaitis M, Felix V, Feng G, Kibbe WA (2012). Disease ontology: a backbone for disease semantic integration. Nucleic Acids Research.

[ref-77] Sergieh HM, Botschen T, Gurevych I, Roth S (2018). A multimodal translation-based approach for knowledge graph representation learning.

[ref-78] Shang C, Tang Y, Huang J, Bi J, He X, Zhou B (2019). End-to-end structure-aware convolutional networks for knowledge base completion. Proceedings of the AAAI Conference on Artificial Intelligence.

[ref-79] Socher R, Chen D, Manning CD, Ng A (2013). Reasoning with neural tensor networks for knowledge base completion.

[ref-80] Su C, Tong J, Zhu Y, Cui P, Wang F (2020). Network embedding in biomedical data science. Briefings in Bioinformatics.

[ref-81] Sun Z, Deng Z-H, Nie J-Y, Tang J (2018). Rotate: knowledge graph embedding by relational rotation in complex space.

[ref-82] Szklarczyk D, Franceschini A, Wyder S, Forslund K, Heller D, Huerta-Cepas J, Simonovic M, Roth A, Santos A, Tsafou KP, Kuhn M, Bork P, Jensen LJ, Von Mering C (2015). String v10: protein-protein interaction networks, integrated over the tree of life. Nucleic Acids Research.

[ref-83] Tang X, Chen L, Cui J, Wei B (2019). Knowledge representation learning with entity descriptions, hierarchical types, and textual relations. Information Processing & Management.

[ref-84] Thafar M, Raies AB, Albaradei S, Essack M, Bajic VB (2019). Comparison study of computational prediction tools for drug-target binding affinities. Frontiers in Chemistry.

[ref-85] Thafar MA, Albaradie S, Olayan RS, Ashoor H, Essack M, Bajic VB (2020a). Computational drug-target interaction prediction based on graph embedding and graph mining.

[ref-86] Thafar MA, Olayan RS, Ashoor H, Albaradei S, Bajic VB, Gao X, Gojobori T, Essack M (2020b). Dtigems+: drug-target interaction prediction using graph embedding, graph mining, and similarity-based techniques. Journal of Cheminformatics.

[ref-87] Trouillon T, Welbl J, Riedel S, Gaussier É, Bouchard G (2016). Complex embeddings for simple link prediction.

[ref-88] Tucker LR (1966). Some mathematical notes on three-mode factor analysis. Psychometrika.

[ref-89] Vashishth S, Sanyal S, Nitin V, Talukdar P (2019). Composition-based multi-relational graph convolutional networks.

[ref-90] Wang H, Xiong W, Yu M, Guo X, Chang S, Wang WY (2019a). Meta reasoning over knowledge graphs. https://arxiv.org/abs/1908.04877.

[ref-91] Wang M, Rong E, Zhuo H, Zhu H (2018). Embedding knowledge graphs based on transitivity and asymmetry of rules.

[ref-92] Wang M, Zheng D, Ye Z, Gan Q, Li M, Song X, Zhou J, Ma C, Yu L, Gai Y, Xiao T, He T, Karypis G, Li J, Zhang Z (2019b). Deep graph library: a graph-centric, highly-performant package for graph neural networks. https://arxiv.org/abs/1909.01315.

[ref-93] Wang Q, Liu J, Luo Y, Wang B, Lin C-Y (2016). Knowledge base completion via coupled path ranking. Proceedings of the 54th Annual Meeting of the Association for Computational Linguistics (Volume 1: Long Papers).

[ref-94] Wang Q, Mao Z, Wang B, Guo L (2017). Knowledge graph embedding: a survey of approaches and applications. IEEE Transactions on Knowledge and Data Engineering.

[ref-95] Wang Q, Wang B, Guo L (2015). Knowledge base completion using embeddings and rules.

[ref-96] Wang Z, Zhang J, Feng J, Chen Z (2014). Knowledge graph and text jointly embedding.

[ref-98] Xie R, Liu Z, Jia J, Luan H, Sun M (2016a). Representation learning of knowledge graphs with entity descriptions.

[ref-99] Xie R, Liu Z, Luan H, Sun M (2016b). Image-embodied knowledge representation learning. https://arxiv.org/abs/1609.07028.

[ref-100] Yanardag P, Vishwanathan S (2015). Deep graph kernels.

[ref-101] Yang B, Yih W-t, He X, Gao J, Deng L (2014). Embedding entities and relations for learning and inference in knowledge bases. https://arxiv.org/abs/1412.6575.

[ref-102] Yue X, Wang Z, Huang J, Parthasarathy S, Moosavinasab S, Huang Y, Lin SM, Zhang W, Zhang P, Sun H (2020). Graph embedding on biomedical networks: methods, applications and evaluations. Bioinformatics.

[ref-103] Zhang L (2002). Knowledge graph theory and structural parsing.

[ref-104] Zhang Z, Zhuang F, Qu M, Lin F, He Q (2018). Knowledge graph embedding with hierarchical relation structure.

[ref-105] Zong N, Kim H, Ngo V, Harismendy O (2017). Deep mining heterogeneous networks of biomedical linked data to predict novel drug-target associations. Bioinformatics.

