# Supplementary material for: Application and evaluation of knowledge graph embeddings in biomedical data
Source: PeerJ Comput Sci. 2021 Feb 18;7:e341. doi: 10.7717/peerj-cs.341 (PMC7959619; doi:10.7717/peerj-cs.341)
Supplement: Supplemental Information 1 [file peerj-cs-07-341-s001.pdf]

Supplementary materials: Application and evaluation of knowledge graph embeddings in biomedical data

Mona Alshahrani, Maha A. Thafar and Magbubah Essack

Appendix A: Macro-AUC, Micro-AUC and F1-score

Figures 1,2, and 3 display the macro-AUC, micro-AUC, and F1-score results. As expected, the *partial* setting results in higher performance compared to the *free* setting across all methods and relation types with Walking RDF and OWL consistently outperforming other methods, with TransE performing comparatively similar. Overall, both methods performed better than Poincare and RESCAL. The *has–indication* relation performed the worst, which is probably because it has the least number of associations (approx. 6,736 retrieved from SIDER). Despite the removal of all relation links in the *free* setting, the performance degradation in the macro-AUC was only limited to 1.63%, 2.85%,8.31% and 3.10% for Walking RDF and OWL, TransE, Poincare, and RESCAL, respectively, in comparison with the *partial* setting. In terms of micro-AUC, the performance decreased by 4.86%, 7.36%, 10.78%, and 11.42% for Walking RDF and OWL, TransE, Poincare, and RESCAL, respectively, compared to the *partial* setting. Furthermore, we used Friedman test to show that there is a statistical significance (with p-value of 2.67×10^-4) in the micro-AUC performance performance by different knowledge graph embeddings methods in the *partial* setting and (with p-value of 7.35×10^-5) in the *free* setting.

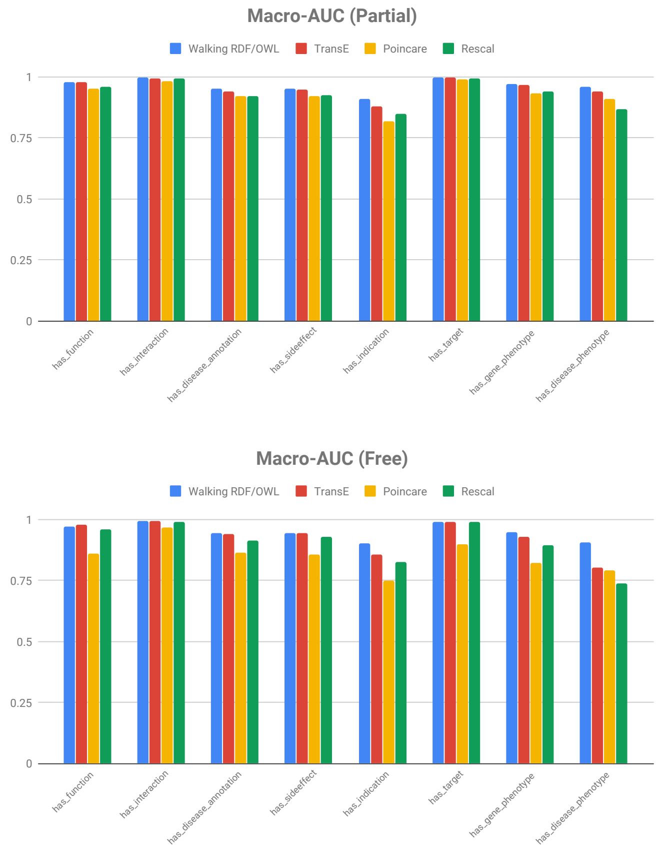

Figure 1: Relation prediction results based on macro-AUC evaluation metric across all methods in the *partial* (top) and *free* (bottom) settings.

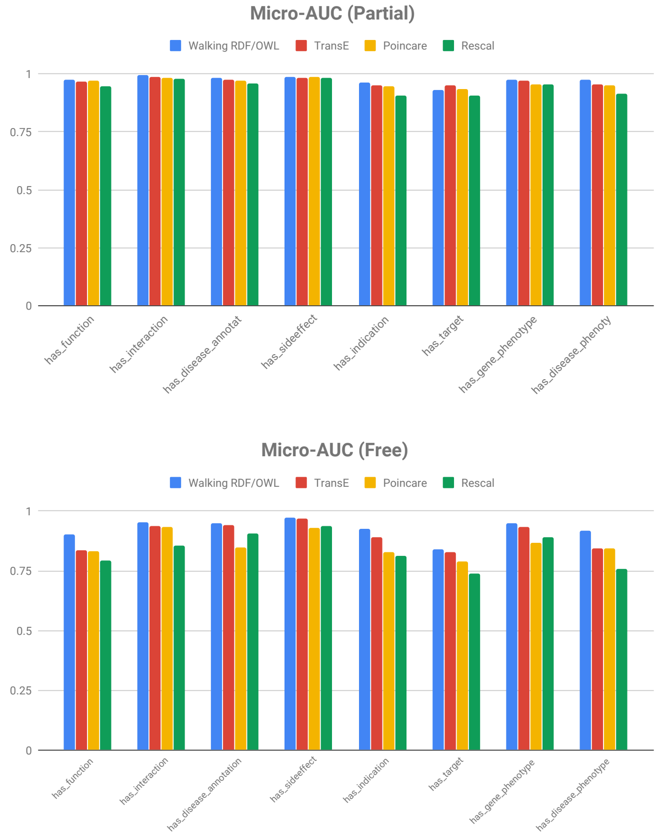

Figure 2: Relation prediction results based on micro-AUC evaluation metric across all methods in the *partial* (top) and *free* (bottom) settings.

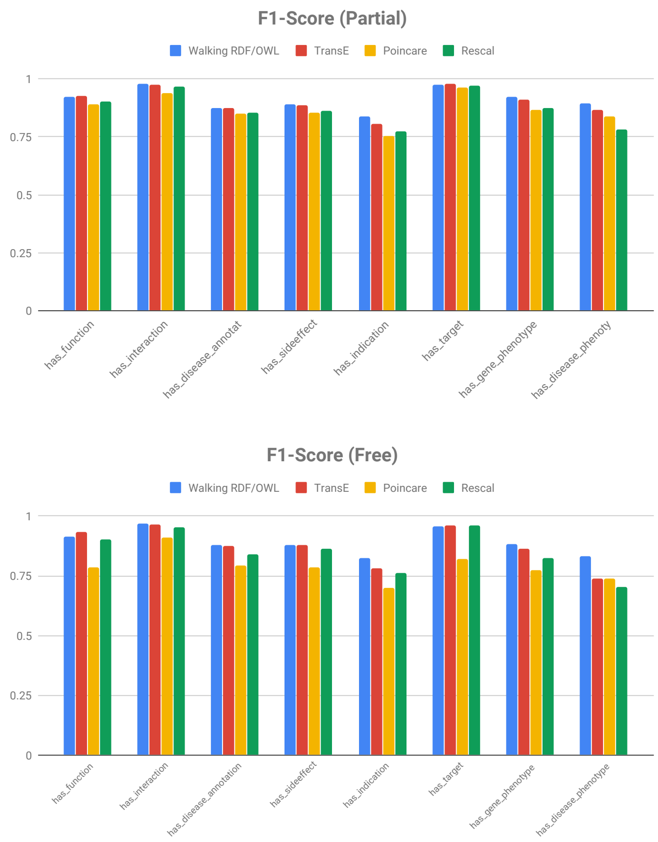

Figure 3: Relation prediction results based on the F1-Score evaluation metric across all methods in *partial* (top) and *free* (bottom) setting
