# Supplementary material for: Application and evaluation of knowledge graph embeddings in biomedical data
Source: PeerJ Comput Sci. 2021 Feb 18;7:e341. doi: 10.7717/peerj-cs.341 (PMC7959619; doi:10.7717/peerj-cs.341)
Supplement: Supplemental Information 2 [file peerj-cs-07-341-s002.pdf]

Supplementary materials: Application and evaluation of knowledge graph embeddings in biomedical data

Mona Alshahrani, Maha A. Thafar and Magbubah Essack

Appendix B1: End-to-end models (native mode)

Table 1 displays the Hit@10 results of relation in the knowledge graph for TransE, Poincare, RESCAL, SimplE and R-GCN. Walking RDF and OWL was developed as a two-stage pipeline described in the (relation prediction section) and therefore cannot be run in an end-to-end manner. We found that the results in this native mode is significantly lower than that of the two-stage pipeline in the case of TransE, but Poincare and RESCAL and SimplE performs better in this mode, specifically for the relations *has–function*, *has–interaction*, and *has-target* (i.e., these are the relations with the largest number of associations). Overall the native mode-related performances were worse for the relations with a lower number of associations. Also, Poincare performed better than TransE and RESCAL in native mode than it did in the two-stage pipeline. This could be due to the way its embeddings were optimized i.e., based on hyperbolic space, (unlike TransE which uses Euclidean space) which may have resulted in features with much more complicated decision boundaries to be learnt by the ANN link prediction model.

Table 1: hits@10 results (*partial* and *free* settings) for relation prediction on our biological knowledge graph as End-to-end models. *partial* and *free* settings are two evaluation strategies correspond to: partially removing 80% of the relation edges and completely removing all relation edges, respectively.

partial					
Relation	TransE	Poincare	Rescal	SimplE	R-GCN
has function	3.528	8.567	6.136	**18.36**	8.57
has interaction	5.277	**18.366**	15.014	7.00	12.49
has disease annotation	14.902	**18.215**	10.178	17.67	17.55
has sideeffect	6.523	6.159	**10.492**	21.60	13.52
has indication	9.319	**9.724**	3.877	12.96	7.86
has target	16.882	**30.800**	18.868	24.37	17.77
has gene phenotype	5.772	4.929	**7.000**	4.30	7.36
has disease phenotype	**12.318**	11.383	6.348	6.49	5.53
free					
Relation	TransE	Poincare	Rescal	SimplE	R-GCN
has function	0.006	0.001	**0.0921**	0.09	0.10
has interaction	4.551	**6.354**	2.442	3.01	1.11
has disease annotation	2.230	**7.188**	0.394	0.32	0.17
has sideeffect	**1.197**	0.453	0.050	0.00	0.02
has indication	0.568	**2.030**	0.081	0.08	0.41
has target	**0.048**	0.039	0.029	0.05	0.00
has gene phenotype	0.577	**0.642**	0.137	0.03	0.11
has disease phenotype	0.077	**1.509**	0.137	0.08	0.04

Appendix B2: Mean rank results

Tables 2 displays the mean rank results of the methods trained as features generators models of relations prediction in the knowledge graph. In the *partial* and *ree* setting, Walking RDF/OWL and SimplE methods perform best, followed by TransE, Poincaré, RESCAL, and RGCN, respectively. Table 3 shows the mean rank performance in the models trained as End-to-end models.

Table 2: The mean rank results (*partial* and *free* settings) for relation prediction of our biological knowledge graph as feature generators models.*partial* and *free* settings are two evaluation strategies correspond to: partially removing 80% of the relation edges and completely removing all relation edges, respectively

			partial			
Relation	Walking RDF/OWL	TransE	Poincaré	Rescal	SimplE	R-GCN
has function	1172	1541	1511	2753	**1138**	1957
has interaction	**166**	241	391	401	249	245
has disease annotation	**191**	261	309	498	211	324
has sideeffect	170	192	201	274	**142**	153
has indication	434	474	598	983	**340**	555
has target	1362	863	1366	1895	**561**	1583
has gene phenotype	**340**	374	579	616	536	493
has disease phenotype	**328**	647	660	1188	750	924
			free			
Relation	Walking RDF/OWL	TransE	Poincaré	Rescal	SimplE	R-GCN
has function	**4829**	7746	8027	10243	6921	11563
has disease annotation	**589**	711	1948	1056	940	732
has sideeffect	**352**	444	1063	951	740	695
has indication	**964**	1038	2398	2267	1530	938
has target	3355	3558	3867	4461	**2945**	3382
has gene phenotype	**724**	939	1794	1498	1544	1989
has disease phenotype	**1077**	2095	2000	3262	2250	3138

Table 3: The mean ranks results (*partial* and *free* settings) for relation prediction on our biological knowledge graph as End-to-end models.*partial* and *free* settings are two evaluation strategies correspond to: partially removing 80% of the relation edges and completely removing all relation edges, respectively

		partial			
Relation	TransE	Poincare	Rescal	SimplE	R-GCN
has function	12478	1802	3323	**1187**	2527
has interaction	977	658	**391**	275	234
has disease annotation	406	**352**	694	245	409
has sideeffect	471	489	**310**	115	162
has indication	702	**502**	686	318	588
has target	1550	1136	**1022**	475	970
has gene phenotype	**747**	997	830	635	622
has disease phenotype	**999**	1056	1436	740	1037
		free			
Relation	TransE	Poincare	Rescal	SimplE	R-GCN
has function	23783	**20075**	25409	32959	21924
has interaction	**2486**	4189	3608	2530	4516
has disease annotation	3071	**2323**	5651	6319	4157
has sideeffect	2786	**1843**	7894	9816	4928
has indication	3078	**1878**	6810	9847	2974
has target	**7387**	8286	9153	12079	11336
has gene phenotype	4882	**2823**	6888	10905	6539
has disease phenotype	7298	**3184**	7260	8704	8991

Appendix B3: Results on Hetionet dataset

Table 4: The mean rank results for relation prediction on the subset of Hetionet dataset as feature generators models.

Relation	Walking RDF/OWL	TransE	Poincaré	Rescal	SimplE	RGCN
treats relation	18	17	50	26	26	**12**
presents relation	**61**	73	97	71	108	78
associates relation	**953**	1915	1041	1829	2091	1773
causes relation	**289**	319	375	362	327	359

Table 5: The mean rank results for relation prediction on the subset of Hetionet dataset as End-to-end models.

Relation	TransE	Poincaré	Rescal	SimplE	RGCN
treats relation	20	29	19	27	18
presents relation	108	94	98	112	82
associates relation	2162	1321	2035	2090	1604
causes relation	448	471	521	351	326

Table 6: The top hit@10 results for relation prediction on the subset of Hetionet dataset as End-to-end models.

Relation	TransE	Poincaré	Rescal	SimplE	R-GCN
treats relation	48.34	26.20	58.94	35.76	56.95
presents relation	16.22	18.29	19.04	11.45	20.53
associates relation	4.63	3.54	2.17	0.35	2.29
causes relation	12.08	3.62	7.25	11.52	7.44
