# Supplementary material for: Application and evaluation of knowledge graph embeddings in biomedical data
Source: PeerJ Comput Sci. 2021 Feb 18;7:e341. doi: 10.7717/peerj-cs.341 (PMC7959619; doi:10.7717/peerj-cs.341)
Supplement: Supplemental Information 3 [file peerj-cs-07-341-s003.pdf]

Supplementary materials: Application and evaluation of knowledge graph embeddings in biomedical data

Mona Alshahrani, Maha A. Thafar and Magbubah Essack

Appendix C: Hyperparameters sensitivity

Additionally, we have conducted several experiments to show the effect of different parameters settings on the output of each method, Figure 1. For this reason, we identified the number of dimensions (dim) in the range of values $[32, 64, 128, 256]$, learning rate (lr) in the range of values $[0.001, 0.01, 0.1, 0.5]$, number of epochs in the range of values $50, 100, 150, 200$ and the number of minibatch with the values $[50, 100, 150, 200]$. These four hyperparameters are common among all of the knowledge graphs methods and are known to be used for parameter optimization (Bordes et al.,2013, Lin et al.,2015). We found that walking-based method is more robust to hyperparameters variations, while transE and Poincare are less sensitive. Rescal seems to perform the worst in this regard, Figure 1.

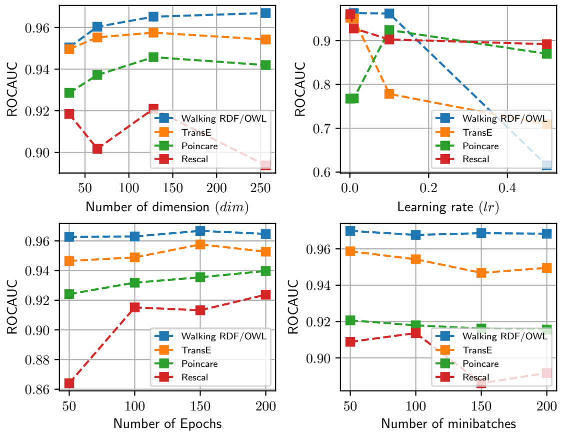

Figure 1: Parameters sensitivity effects using various different setting with each method.
