# Supplementary material for: Application and evaluation of knowledge graph embeddings in biomedical data
Source: PeerJ Comput Sci. 2021 Feb 18;7:e341. doi: 10.7717/peerj-cs.341 (PMC7959619; doi:10.7717/peerj-cs.341)
Supplement: Supplemental Information 4 [file peerj-cs-07-341-s004.pdf]

# Supplementary materials: Datasets

Mona Alshahrani, Maha A. Thafar and Magbubah Essack

## Appendix D: Datasets information

Table 1: Number of relation edges and participating entities in each relation of our biological knowledge graph

| Relation | # of relation edges | # of source entities | # of destination entities |
|---|---|---|---|
| has function | 244,105 | 16,629 (Entrez Gene) | 17,218 (Gene ontology) |
| has interaction | 240,775 | 7,956 | 8,239 |
| has disease annotation | 29,984 | 6,619 | 1,767 |
| has sideeffect | 81,006 | 1,421 | 1,413 |
| has indication | 6,190 | 1,229 | 875 |
| has target | 432,512 | 98,567 | 9,782 |
| has gene phenotype | 153,575 | 3,526 | 6,058 |
| has disease phenotype | 84,508 | 6,144 | 6,000 |

Table 2: Number of relation edges and participating entities in each relation of subset of Hetionet dataset

| Relation | # of relation edges | # of source entities | # of destination entities |
|---|---|---|---|
| treats relation | 755 | 387 (Drugbank) | 77 (Disease ontology) |
| presents relation | 3,357 | 133 (Disease ontology) | 415 (MeSH) |
| associates relation | 12,623 | 134(Disease ontology) | 5,392 (Entrez Gene) |
| causes relation | 138,944 | 1,071 (Drugbank) | 5,701 (SIDER) |
